# Objective sleep was longitudinally associated with brain amyloid burden in mild cognitive impairment

**DOI:** 10.1002/acn3.51912

**Published:** 2023-09-30

**Authors:** Noriyuki Kimura, Yuuki Sasaki, Teruaki Masuda, Takuya Ataka, Atsuko Eguchi, Tatsuyuki Kakuma, Etsuro Matsubara

**Affiliations:** ^1^ Department of Neurology, Faculty of Medicine Oita University Oita Japan; ^2^ Biostatistics Center Kurume University Kurume Japan

## Abstract

**Objective:**

Understanding the longitudinal association of objective sleep and physical activity with brain amyloid burden and cortical glucose metabolism has critical clinical and public health implications for dementia prevention in later life.

**Methods:**

We enrolled 118 individuals aged ≥65 years with mild cognitive impairment, who were followed up on from August 2015 to September 2019. All participants continuously wore an accelerometer sensor for 7 consecutive days every 3 months and received annual ^11^C‐Pittsburgh compound‐B and ^18^F‐fluorodeoxyglucose positron emission tomography (PET). Sleep and physical activity parameters were assessed using accelerometer sensor data and PET imaging was quantified using a standardized uptake‐value ratio. Fifty‐seven participants (48.3%) completed a lifestyle factor assessment and PET imaging over the 3‐year period. A linear mixed‐effects model was applied to examine the longitudinal association of sleep and physical activity parameters with PET imaging over the 3‐year period, controlling for potential confounders.

**Results:**

Sleep efficiency was inversely associated with amyloid uptake in the frontal lobe. Although sleep duration was positively associated with global amyloid uptake, particularly in the frontal lobe, their impact was extremely small. However, physical activity parameters were not significantly associated with the ^11^C‐Pittsburgh compound‐B‐uptake. Furthermore, sleep and physical activity parameters were not significantly associated with cortical glucose metabolism.

**Interpretation:**

Lower sleep efficiency could be an early symptom of greater brain amyloid burden at the mild cognitive impairment stage. Therefore, the assessment of sleep may be useful for identifying individuals at higher risk for brain amyloid burden. Future longer term observational studies are required to confirm these findings.

## Introduction

Mild cognitive impairment (MCI) is a high‐risk factor for the progression to dementia, and the rate of conversion from amnestic MCI to Alzheimer's disease (AD) is 10–15% per year.^1^ In Japan, the prevalence of MCI among people aged 65 years and older is 17.0%.^2^ AD is an important public health problem with a tremendous emotional and financial burden on patients, caregivers, and society. Although disease‐modifying therapy (lecanemab) is emerging, efficacy is limited and side effects cannot be ignored.^3^ Therefore, determining the modifiable risk factors of AD is critical in reducing the risk of AD incidence or delaying the onset of AD at the MCI stage. Previous epidemiological studies have reported the association of lower educational levels, vascular risk factors, depression, and smoking with an increased risk of AD.^4^, ^5^

However, sleep disorders and physical inactivity are also major issues in the rapidly aging society. In fact, approximately 50% of older adults have sleep problems and 12.5–27.5% of older adults are physically inactive.^6^, ^7^, ^8^ Previous population‐based prospective studies and meta‐analyses have shown that these inevitable changes were associated with subsequent cognitive decline or an increased risk of AD in older adults.^9^, ^10^, ^11^ Although studies of human and animal models of AD suggest that sleep–wake cycle and physical activity may affect the production or clearance of amyloid β (Aβ),^12^, ^13^ it remains unclear whether sleep disturbance and physical inactivity are early symptoms or causes of AD pathology. This is because most of the population‐based prospective studies of the association of subjective or objective sleep and physical activity with brain amyloid burden had cross‐sectional designs.^14^, ^15^, ^16^, ^17^, ^18^, ^19^, ^20^, ^21^, ^22^, ^23^, ^24^, ^25^, ^26^, ^27^, ^28^, ^29^, ^30^, ^31^, ^32^, ^33^, ^34^ As the sleep–wake cycle, physical activity, and brain amyloid burden change with aging,^35^, ^36^, ^37^ longitudinal studies of the association between the trajectories of sleep, physical activity, and amyloid biomarkers over time would provide more valuable evidence and dynamic perspectives on their associations than cross‐sectional studies. Moreover, subjective self‐reported questionnaires may often be problematic with regard to their reliability and consistency due to recall bias or misclassification among older adults with cognitive decline. In contrast, an accelerometer sensor is a noninvasive and cost‐effective tool that can objectively and continuously measure sleep parameters and physical activity without recall bias or misclassification.^38^, ^39^

In this prospective cohort study, we objectively measured lifestyle factors using accelerometer sensors and evaluated the brain amyloid burden and cortical glucose metabolism using ^11^C‐Pittsburgh compound‐B positron emission tomography (PiB‐PET) and ^18^F‐fluorodeoxyglucose (FDG)‐PET, respectively, in older community‐dwelling Japanese individuals with MCI from 2015 to 2019. To the best of our knowledge, few other cohort studies have focused on the longitudinal association of sleep or physical activity with AD biomarkers in older individuals. Therefore, this study aimed to determine the longitudinal association between objective sleep and physical activity based on accelerometer sensor data, brain amyloid burden, and cortical glucose metabolism over a 3‐year period in older individuals with MCI. We hypothesized that the sleep or physical activity parameters would be longitudinally associated with PiB‐uptake over time.

## Materials and Methods

### Participants

**Figure 1:**
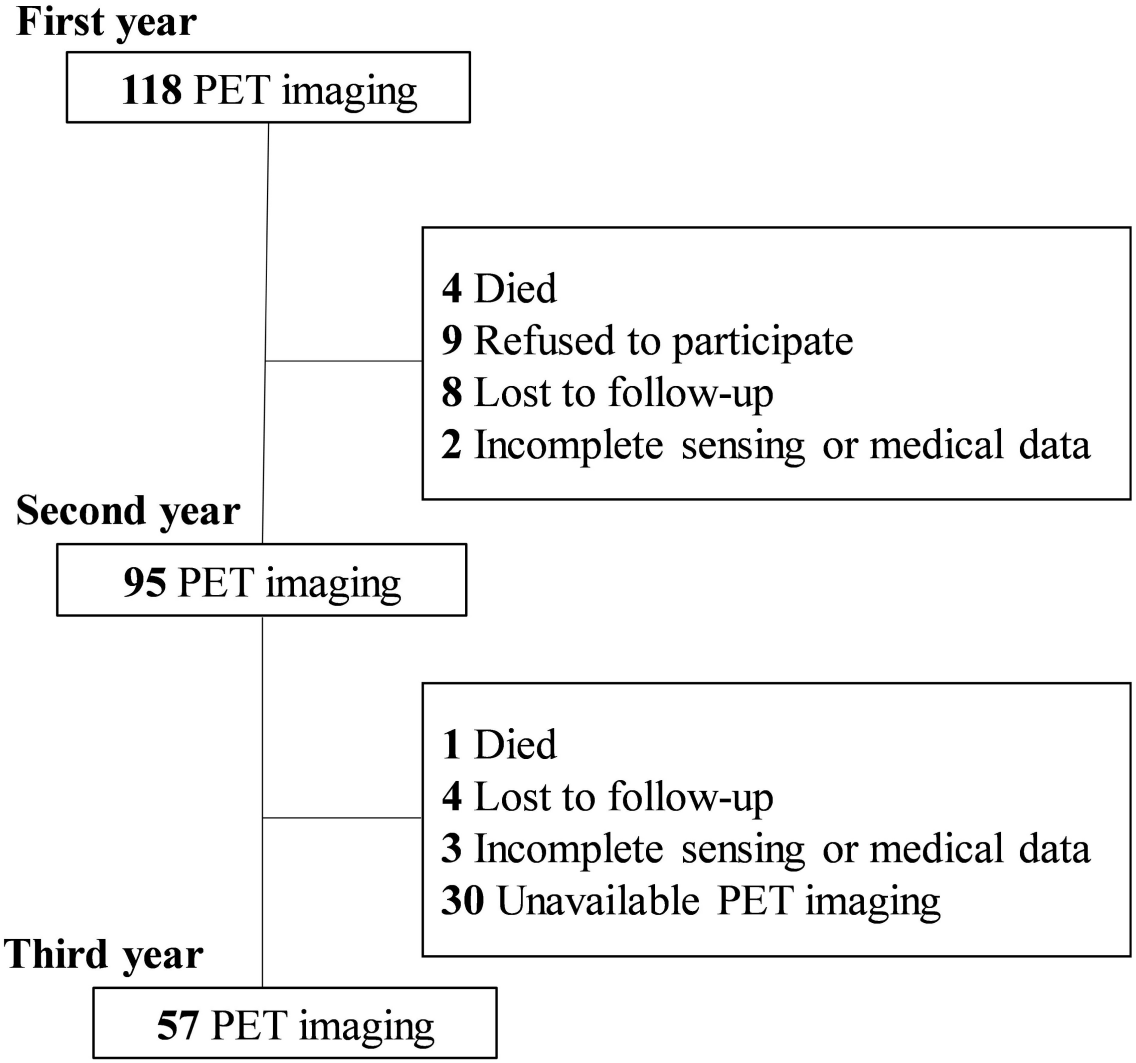
The flow of participant recruitment. One‐hundred‐and‐eighteen participants with mild cognitive impairment underwent PET imaging in the first year. Subsequently, 95 (80.5%) and 57 (48.3%) participants with MCI underwent PET imaging in the second and third years, respectively. MCI, mild cognitive impairment; PET, positron emission tomography.

The Usuki study was a prospective cohort study of community‐dwelling adults aged ≥65 years without dementia.^38^, ^39^ This was conducted from August 2015 to September 2019 in Usuki, Oita Prefecture, Japan, and it explored lifestyle risk factors for dementia. In the present study, we included 118 adults with MCI [52 men (44.1%) and 66 women (55.9%), with a mean age of 75.7 years (standard deviation [SD], 5.8) and a mean educational level of 11.2 years (SD, 1.9)] who were enrolled in the imaging process of the Usuki study. All participants wore a wristband sensor (Silmee™ W20, TDK Corporation, Tokyo, Japan) continuously—except for when bathing—for 7 consecutive days every 3 months (four times per year) to avoid measurement errors due to seasonal differences in sleep and activity patterns.^40^ Valid sensing data was defined as at least 3 days in one period and two periods in a year.^41^ In addition, PiB‐ and FDG‐PET scans and demographic data collection, including that of age, sex, educational level, medication history, and dementia diagnosis, were conducted every year by trained medical staff. The number of adults with MCI from whom both valid accelerometer sensor data and PET imaging were completely collected during the follow‐up was 95 (80.5%) in the second year and 57 (48.3%) in the third year (Fig. 1). The diagnosis of MCI was made when participants had a Clinical Dementia Rating score of 0.5 and unimpaired activities of daily living.^42^ Furthermore, all the participants had Hachinski ischemic scores of 4 or less.^43^ We excluded participants with a history of other neurological or psychiatric disorders, severe cardiac failure, severe hepatic or renal dysfunction, severe head trauma, alcoholism, and stroke, as well as those undergoing treatment for cancer.

### Accelerometer sensor data

Sleep and physical activity were calculated using the sum of the sensor data per day. Sleep parameters included the total sleep time (TST), sleep efficiency, waking frequency, and time awake after sleep onset (WASO). The time of sleep onset was determined as the beginning timepoint of the first continuous block of 20 min of sleep without movement. The TST was defined as the sum of minutes without more than 5 min of movement from sleep onset to the end of sleep. Moreover, nocturnal awaking was defined as a continuous block of 5–90 min of movement after sleep onset. Sleep efficiency was defined as the percentage of TST in the total time in bed, while WASO was calculated as the total amount of time spent awake after sleep onset. Furthermore, naptime was calculated from the time spent resting without moving during the daytime.

In addition, physical activity was assessed continuously by a three‐axis accelerometer, and the data were summarized in 1‐min intervals. Steps were defined as the frequency range of 2–3 Hz of acceleration. Additionally, based on metabolic equivalents (METs), the intensity of activity was divided into light physical activity (LPA, 1.6–2.9 METs), moderate‐to‐vigorous physical activity (MVPA, ≥3.0 METs), and sedentary behavior (≤1.5 METs), as previously described.^44^ The total amount of time spent in each physical activity was measured when the participants were awake. However, data (according to the heart rate) indicating that the wristband sensor had been removed were excluded. The measurement accuracy for sleep time and walking steps was verified using the comparison between the sensor data and video observations in healthy individuals aged 20–60 years.^38^ Notably, the sleep duration and walking steps from the wristband sensor significantly correlated to those from video observation (Pearson correlation: *r* = 0.9995 and *r* = 0.9869, respectively).

### Positron emission tomography scans

PET images were acquired in the three‐dimensional scanning mode on a Siemens Biograph mCT PET scanner (Siemens, Erlangen, Germany). All participants underwent a rapid bolus intravenous injection of ^11^C‐PiB (mean, 548 MBq; SD, 53 MBq) with a saline flush and received static PET scanning over 50–70 min after injection. Additionally, all participants underwent a rapid bolus intravenous injection of ^18^F‐FDG (mean, 170 MBq; SD, 30 MBq) with a saline flush and received static PET scanning over 40–60 min after injection. Subsequently, spatial normalization of both the PiB and FDG scans to a customized PET template in the Montreal Neurological Institute reference space was conducted using Statistical Parametric Mapping Version 8 (Wellcome Trust Center for Neuroimaging, London, UK). Furthermore, the MarsBaR toolbox for Statistical Parametric Mapping (MRC Cognition and Brain Sciences Unit, Cambridge, UK) was used to set the regions of interest (ROIs) for characteristic amyloid burden or hypometabolism areas in patients with AD.^45^, ^46^ These ROIs included the frontal lobe, temporoparietal lobe, and posterior cingulate gyrus.

Both PiB‐ and FDG‐uptake were measured quantitatively using a standardized uptake‐value ratio (SUVR). The regional PiB‐ and FDG‐PET SUVRs were calculated as the ratio of the voxel number‐weighted average of the median uptake in each ROI to that in the cerebellar cortex. In addition, the global FDG‐ and PiB‐PET SUVRs were computed as the single mean value for the regional SUVRs across a set of ROIs. Global cortical SUVR values ≥1.4 were considered positive for PiB.

### Apolipoprotein E phenotype

The Apolipoprotein E (ApoE) phenotype was examined using the enzyme‐linked immunosorbent assay kit for human apolipoprotein E4/Pan‐ApoE (MBL Co., Ltd., Woburn, USA). This sandwich ELISA kit can quantify the amount of ApoE4 or total ApoE using an affinity‐purified polyclonal antibody targeting *ApoE* and monoclonal antibody targeting *APOE4*. According to previous methods, we defined the homozygote (ε4/ε4) or heterozygote (ε2/ε4, ε3/ε4) *APoE4* phenotype as a ratio of ApoE4 and ApoE of 0.3 or higher.^47^

### Statistical analysis

Our data analysis primarily aimed to examine the dynamic association of sleep and physical activity with brain amyloid burden or cortical glucose metabolism over a 3‐year follow‐up period in all participants who completed both valid accelerometer sensor data and PET imaging. We used a linear mixed‐effects model to determine whether the sleep and physical activity parameters were longitudinally associated with the global or regional uptake for both PiB‐ and FDG‐PET after controlling for age, sex, educational level, and ApoE4 status. This model controls for the dependency between repeated measurements of the same participants, the initial level of cognitive function, and missing values. The effect of the follow‐up time was modeled as discrete, and the interaction between time‐varying sleep and physical activity parameters and follow‐up time was not included in the model due to clinical and statistical difficulties in parameter interpretation. In addition, a compound symmetry structure was specified for within‐subject serial correlation among repeated measures of PiB‐ and FDG‐PET imaging by including a random‐intercept term in the model. Overfitting of the model was controlled using the Akaike Information Criterion. We used the JMP Pro 14.2.0 (SAS Institute Japan Ltd., Tokyo, Japan) and IBM Statistical Package for the Social Sciences Statistics Version 25.0 (IBM Corp., Armonk, NY, USA) for statistical analyses, and a *p*‐value <0.05 was considered to be statistically significant.

### Ethics

This study was conducted following the Declaration of Helsinki and was approved by the local ethics committee (study approval numbers: UMIN000017442). All participants provided written informed consent. This research complies with the Strengthening the Reporting of Observational Studies in Epidemiology reporting guideline.

## Results

### Demographic and clinical characteristics

Table 1 summarizes the annual changes in demographic characteristics and PiB‐ and FDG‐uptake values of all participants. In total, 17 out of 118 (14.4%) individuals had *ApoE4* and 27 (22.9%) were included in the greater PiB‐uptake group according to a PiB‐PET SUVR cutoff of 1.4. Table 2 summarizes the annual changes in accelerometer sensor data.

**Table 1:** Clinical and demographic characteristics of participants with mild cognitive impairment.

Characteristics	First year (*n* = 118)	Second year (*n* = 95)	Third year (*n* = 57)
	Mean (SD)	Mean (SD)	Mean (SD)
Age, years	75.7 (5.8)	76.2 (5.5)	75.8 (5.7)
Sex (M:W)	52:66	42:53	28:29
Educational level, years	11.2 (1.9)	11.2 (1.8)	11.5 (1.9)
BMI, kg/m^2^	23.2 (3.3)	23.6 (3.2)	23.6 (3.1)
*ApoE4*, *n* (%)	17 (14.4)	14 (14.7)	12 (21.1)
Global PiB‐uptake	1.16 (0.51)	1.16 (0.52)	1.19 (0.56)
Global FDG‐uptake	0.92 (0.08)	0.92 (0.08)	0.91 (0.08)

ApoE4, apolipoprotein ε4; BMI, body mass index; FDG, ^18^F‐fluorodeoxyglucose; M, men; min, minute; PiB, ^11^C‐Pittsburgh compound‐B; SD, standard deviation; W, women.

**Table 2:** Wearable sensor data of participants with mild cognitive impairment.

Characteristics	First year (*n* = 118)	Second year (*n* = 95)	Third year (*n* = 57)
	Mean (SD)	Mean (SD)	Mean (SD)
TST, min/day	404.9 (68)	413.4 (73)	412.2 (76.5)
Sleep efficiency, %/day	95.1 (3.6)	95.2 (3.7)	95.3 (3.4)
Waking frequency, times/day	0.52 (0.36)	0.52 (0.38)	0.49 (0.32)
WASO, min/day	20.2 (14.5)	20.6 (15.9)	19.7 (13.5)
Naptime, min/day	43.1 (32.3)	45 (35.6)	49.5 (38.7)
Walking steps, steps/day	4619.7 (2726.7)	4438.4 (2781.8)	3942.4 (2288.5)
LPA, min/day	20.4 (16.6)	20.8 (15.3)	18.3 (11.9)
MVPA, min/day	23.7 (18)	21.9 (18.8)	19.9 (15.1)
Sedentary, min/day	785.6 (74.9)	780.8 (75.8)	771 (80.9)

LPA, light physical activity; min, minute; MVPA, moderate‐to‐vigorous physical activity; SD; standard deviation; TST, total sleep time; WASO, time awake after sleep.

### Longitudinal association between sleep and physical activity parameters with PiB‐ and FDG‐uptake

TST was positively associated with the global and frontal PiB‐uptake after controlling for age, sex, educational level, and *ApoE4* status (Table 3) (global: estimate, 0.00048; standard error, 0.00022; *P* = 0.0332; frontal: estimate, 0.00053; standard error, 0.00022; *P* = 0.017). Moreover, sleep efficiency (%) was inversely associated with the frontal PiB‐uptake (Table 3) (estimate, −0.02116; standard error, 0.00944; *P* = 0.0267). These results indicate that a one minute increase in TST would be associated with an increase of 0.00048 in global PiB SUVR or 0.00053 in frontal PiB SUVR and a 10% increase in sleep efficiency would be associated with −0.2116 in frontal PiB SUVR. When the linear mixed‐effects model was conducted using data from 57 adults who had completely valid wearable sensor and PET imaging data for 3 years, it was found that TST remained positively associated with the global and frontal PiB‐uptake (global: estimate, 0.00057; standard error, 0.00029; *P* = 0.049; frontal: estimate, 0.00065; standard error, 0.00028; *P* = 0.0255). However, other sleep parameters—such as waking frequency, WASO, and naptime—and physical activity parameters were not significantly associated with brain amyloid burden. Furthermore, there was no significant association of sleep and physical activity parameters with global and regional FDG‐uptake.

**Table 3:** Linear mixed‐effects models estimating the longitudinal associations of sleep and physical activity with PET imaging.

	Total sleep time			Sleep efficiency		
	Estimate	SE	*P*‐value	Estimate	SE	*P*‐value
PiB‐uptake						
Global	0.00048	0.00022	0.0332*	−0.01717	0.00947	0.072
Frontal lobe	0.00053	0.00022	0.017*	−0.02116	0.00944	0.0267*
Temporoparietal lobe	0.00034	0.00022	0.1348	−0.0108	0.0096	0.2626
Posterior cingulate gyrus	0.00054	0.00031	0.0774	−0.01211	0.01309	0.3566

Adjustments were made for age, sex, educational level, and *ApoE4* status.

PET, positron emission tomography; PiB, ^11^C‐Pittsburgh compound‐B; SE, standard error.

* *P* < 0.05.

## Discussion

The present study examined the longitudinal association of the objectively and simultaneously measured sleep and physical activity parameters with brain amyloid burden over a 3‐year follow‐up period. We found that a longer sleep duration and lower sleep efficiency was significantly associated with greater brain amyloid burden, particularly in the frontal lobe at the MCI stage. These novel and interesting findings are valuable as they have the potential to clarify the relationship between sleep and AD pathology in older individuals. Moreover, the several strengths of this study include the prospective population‐based cohort, the objective measurement of lifestyle factors every 3 months, and the performance of PET imaging every year over the 3‐year follow‐up.

This longitudinal objective analysis showed that TST was positively associated with the global and frontal PiB‐uptake and sleep efficiency was inversely associated with the frontal PiB‐uptake over 3 years. Notably, a previous study showed that sleep disturbances affect 25–40% of patients with AD and are bidirectionally linked to AD pathology.^11^ Given the prolonged period of Aβ accumulation during the asymptomatic preclinical phase, sleep disturbances are assumed to be either an early symptom of AD pathology and/or a risk factor for AD pathology.^12^ Several studies have examined the association of subjective or objective sleep parameters with brain amyloid burden using amyloid PET imaging or the Aβ42 level in the cerebrospinal fluid in cognitively healthy adults.^14^, ^15^, ^16^, ^17^, ^18^, ^19^, ^20^, ^21^, ^22^, ^23^, ^24^, ^25^, ^26^, ^27^ Furthermore, the results of subjective analysis using self‐reported questionnaires have shown that shorter or longer sleep durations, longer sleep latency, lower sleep quality, excessive daytime sleep or somnolence, and more sleep problems are associated with greater brain amyloid burden.^14^, ^15^, ^16^, ^17^, ^18^, ^19^, ^20^, ^21^ Similarly, the results of objective analysis have shown that increased sleep latency, sleep fragmentation, lower sleep efficiency on wearable sensors, and disruption of slow‐wave activity on polysomnography are associated with greater brain amyloid burden.^22^, ^23^, ^24^, ^25^, ^26^, ^27^ However, these studies only assessed sleep parameters and brain amyloid burden at baseline. One study has reported that low sleep efficiency and decreased slow‐wave activity on polysomnography at baseline were longitudinally associated with increased brain amyloid burden, whereas the association of TST or WASO with increased brain amyloid burden over time was not significant.^48^ In contrast, the current study assessed both objective sleep parameters and brain amyloid burden over a 3‐year period.

We found the association of an increase of one minute in TST with an increase of 0.00048 in global PiB SUVR or 0.00053 in frontal PiB SUVR, and the association of a 10% increase in sleep efficiency with −0.2116 in frontal PiB SUVR. The finding of an association between lower sleep efficiency and greater brain amyloid burden in this study was consistent with those of previous objective studies. Moreover, considering that the annual increase in brain amyloid burden is estimated to be 0.043 SUVR in amyloid‐positive adults with normal cognition,^49^ a few percent improvement in sleep efficiency would be equivalent to 1 year of brain amyloid burden. Although we found that a longer sleep duration was associated with greater brain amyloid burden in all the participants as well as 57 participants who completed a lifestyle factor assessment and annual PET, the impact of TST on brain amyloid burden was negligible. We suggest that the impact of sleep efficiency on brain amyloid burden may be more robust compared to sleep duration. However, the clinical relevance of increased brain amyloid burden due to lower sleep efficiency should be interpreted with caution. The causal relationship between sleep efficiency and brain amyloid burden remains unclear. Moreover, Aβ aggregation in the brain occurs 20 years before dementia onset^50^ and PiB‐uptake only weakly correlates with cognitive tests.^51^ However, lower sleep efficiency may be associated with clinical outcome in adults with MCI through brain amyloid burden because of the association between increased brain amyloid burden and a higher risk of developing dementia.^37^ Further longitudinal studies are required to confirm whether these effect the amount of sleep efficiency or sleep duration on the brain amyloid burden in this study that is meaningful for cognitive function.

Previously, potential mechanisms linking sleep disturbance and brain amyloid burden have been identified in human and animal models of AD.^52^, ^53^ Notably, AD mouse models and older adults with brain amyloid burden had the disruption of physiological fluctuations in amyloid β.^52^ Additionally, acute sleep deprivation increased the interstitial fluid levels of soluble amyloid β, leading to amyloid plaque formation in both the mouse model of AD and the human brain.^52^, ^53^ Therefore, these mechanisms may explain the association between a lower sleep efficiency and greater brain amyloid burden in this study. Similar to our findings, a previous study showed that an increased sleep duration (from 7–8 to ≥9 h) over 8.5 years was associated with an increased risk for cognitive decline.^54^ Furthermore, another study showed that a longer sleep duration ≥9 h was associated with a higher risk of dementia‐specific mortality.^55^ These studies suggest that a sleep duration of longer than 7 h per night may be an early symptom of dementia. Moreover, prolonged sleep was considered as a marker of neurodegeneration based on the finding of an association between prolonged sleep and total brain volume.^56^ Therefore, our findings support the hypothesis that a longer sleep duration is consequences of the AD pathology and are potential early symptoms of greater brain amyloid burden at the MCI stage. Another possible mechanism for the association between a longer sleep duration and brain amyloid burden is underlying health problems. Chronic inflammatory diseases, such as cardiovascular disease and Type‐2 diabetes, were associated with systemic inflammation and sleep disturbances.^57^, ^58^ Moreover, chronic inflammation may contribute to prolonged sleep duration and AD pathology.^59^, ^60^, ^61^, ^62^ Therefore, medical comorbidities may be associated with increased functional disability and sedentary behavior, which can overestimate sleep duration as participants spent more time in bed due to inactivity.

Moreover, in our study, sleep duration and efficiency were associated with brain amyloid burden, particularly in the frontal lobe. Several previous studies assessing brain amyloid burden using amyloid PET studies have shown the association of poor sleep quality or a short sleep duration with greater brain amyloid burden, particularly in the precuneus, frontal lobe, angular gyrus, or cingulate gyrus, which are known to be affected by amyloid β at an early stage of AD.^14^, ^15^, ^16^, ^17^, ^18^ Therefore, our findings are partially consistent with those of previous studies.

However, in this study, we did not find a longitudinal association of objective physical activity parameters with PiB‐ and FDG‐uptake in older adults with MCI. In contrast, several previous cross‐sectional studies of subjective or objective physical activity parameters and brain amyloid burden using amyloid PET imaging or the Aβ42 level in the cerebrospinal fluid^28^, ^29^, ^30^, ^31^, ^32^, ^33^, ^34^ have shown that higher levels of physical activity were associated with less brain amyloid burden in cognitively healthy older adults.^28^, ^29^, ^30^, ^31^ Similar to our study, some investigations have found no significant association between self‐reported physical activity and brain amyloid burden in older individuals with MCI or AD.^32^, ^33^, ^34^ A possible explanation for this discrepancy may be that the association of physical activity with brain amyloid burden may be found only in the preclinical phase. Furthermore, these studies had differing study designs and methods of measuring physical activity.

The present study had several limitations. First, the potential direction of causality could not be determined due to the relatively short follow‐up period. Moreover, the linear mixed‐effects model could not be conducted in the subgroup classified based on the PiB‐uptake values due to its relatively small sample size of MCI adults with greater brain amyloid burden. The PiB‐uptake values were skewed to low amyloid accumulation, indicating early stages of MCI. Second, the 61 participants who could not receive PET imaging in the third year were excluded from the analysis and the individuals with dementia or depression could not be completely excluded based on the collected information of the presence or absence of dementia. Third, we could not verify the measurement accuracy for sleep efficiency and WASO other than sleep time, and non‐rapid eye movement or rapid eye movement sleep stages were not evaluated by polysomnography. Although sleep‐disordered breathing is associated with brain amyloid deposition and dementia, and its prevalence increased with age,^63^, ^64^, ^65^ that was not evaluated in this study. Fourth, global cognitive function generally correlates with tau pathology rather than amyloid pathology in AD^51^. The clinical relevance of increased brain amyloid burden due to lower sleep efficiency or prolonged sleep duration remains unclear.

In conclusion, we confirmed the longitudinal associations of objectively measured sleep duration and efficiency with brain amyloid burden over a 3‐year follow‐up in older individuals with MCI. These results suggest that a lower sleep efficiency could be an early symptom of greater brain amyloid burden in later life. Therefore, the assessment of sleep quality may be a cost‐effective and noninvasive tool for identifying individuals at a higher risk of brain amyloid burden. However, future studies with longer follow‐up periods are required to further clarify the causal relationship between sleep and amyloid pathology. Additionally, examining the influence of the clearance of Aβ on sleep duration or efficiency may provide valuable information for the validation of our results when anti‐amyloid therapy becomes available.

## Author Contributions

Initial drafting of the manuscript was carried out by Noriyuki Kimura. Noriyuki Kimura, Teruaki Masuda, Takuya Ataka, Atsuko Eguchi, and Etsuro Matsubara performed the acquisition, analysis, and interpretation of data. Statistical analyses were performed by Tatsuyuki Kakuma and Yuuki Sasaki. Etsuro Matsubara provided administrative and technical support for this project. All authors reviewed and approved the final manuscript.

## Funding Information

This research was supported by grants from the Japan Agency for Medical Research and Development [Grant Number: 18he1402003] and Grant‐in‐Aid for Scientific Research (C) [Grant Number: 22K07474].

## Conflict of Interest

Dr. Kimura received honorarium from Takeda Pharmaceutical, Daiichi Sankyo, Eisai, Sumitomo Pharma, Kyowa Kirin, and Otsuka Pharmaceutical outside the submitted work. No other disclosures were reported. No other disclosures were reported.

## Consent for Publication

Not applicable in this study.

## Data Availability

The data used in this study cannot be shared publicly due to ethical restrictions. The participants signed an informed consent form, which states that their data are exclusively available for research institutions in an anonymized form. The raw data used in this study contain sensitive and identifying information on individuals, including their sex, age, and education level, which could compromise the privacy of research participants. However, the data that support the findings of this study are available upon ethical approval by the local ethics committee of the Oita University Hospital. Please contact the ethics committee of the Oita University Hospital to obtain this data (Email: rinrikenkyu@oita-u.ac.jp).
